# Genotyping and Antibiotic Resistance Traits in *Campylobacter jejuni* and *coli* From Pigs and Wild Boars in Italy

**DOI:** 10.3389/fcimb.2020.592512

**Published:** 2020-10-15

**Authors:** Francesca Marotta, Lisa Di Marcantonio, Anna Janowicz, Francesca Pedonese, Guido Di Donato, Adrian Ardelean, Roberta Nuvoloni, Elisabetta Di Giannatale, Giuliano Garofolo

**Affiliations:** ^1^ National Reference Laboratory for Campylobacter, Istituto Zooprofilattico Sperimentale dell’Abruzzo e del Molise “G. Caporale”, Teramo, Italy; ^2^ Department of Veterinary Sciences, University of Pisa, Pisa, Italy; ^3^ Institute for Diagnosis and Animal Health, National Reference for TSEs and Anatomic Pathology Laboratory, Bucharest, Romania

**Keywords:** *Campylobacter*, antimicrobial resistance (AMR), multidrug resistance (MDR), multilocus sequence typing, resistance genes, wgMLST

## Abstract

The present study investigated the genomic constitution and antimicrobial resistance (AMR) of 238 *Campylobacter* from pigs and wild boars in Italy between 2012 and 2019. *Campylobacter* strains were genotyped using multilocus sequence typing (MLST) and whole genome MLST (wgMLST), screened for antimicrobial resistance genes, and tested for phenotypic susceptibility to six different antibiotics. *C. coli* was detected in 98.31% and 91.66% of pigs and wild boars, while *C. jejuni* was isolated in the remaining cases. MLST assigned 73 STs and 13 STs in pigs and wild boars, respectively, including 44 novel STs. The predominant ST in pigs was ST-854 (12.36%), followed by ST-9264 (6.18%). ST-1055 and ST-1417 were predominant in wild boars (30% and 13.33%, respectively). The minimum spanning tree using 1,121 global MLST profiles showed specific Italian clusters and a clear separation between pig and wild boar profiles. The wgMLST confirmed the MLST clustering and revealed a high genetic diversity within *C. coli* population in Italy. Minimum inhibitory concentrations (MIC) of six antibiotics revealed higher resistance in pigs to ciprofloxacin, nalidixic acid, streptomycin and tetracycline, compared to wild boar. In contrast, most strains were susceptible to gentamicin. Worrying levels of multidrug resistance (MDR) were observed mostly in pig isolates. Molecular screening of AMR mechanisms revealed the predominance of gyrA T86I substitution among fluoroquinolone- and quinolone-resistant isolates, and the 23S rRNA A2075G mutation among macrolide-resistant isolates. Other resistance determinants were observed: (i) *tet(O)* gene was present among tetracycline-resistant isolates; (ii) *rpsL* and *aph*(3’)-III genes conferring resistance to aminoglycosides, were identified only in streptomycin or gentamicin-resistant pig isolates; (iii) *cmeA, cmeB, cmeC, cmeR* genes responsible of pump efflux mechanisms, were observed in almost all the strains; (iv) *OXA*-61, encoding β-lactamase, was found in the half of the strains. Genotypic and phenotypic AMR profiling was fairly correlated for quinolones/fluoroquinolones. *Campylobacter* infection is common also in wild boar populations in Italy, suggesting that wild boars could be a reservoir of resistant and multi-resistant *Campylobacter* species, which may be of public health concern. The present study adds to our knowledge on the epidemiological and ecological traits of this pathogen in domesticated and wild swine.

## Introduction

*Campylobacter* is known as the most common cause of bacterial gastrointestinal infection in Europe, with the annual number of cases exceeding those of salmonellosis and shigellosis (EFSA & ECDC, 2019). *Campylobacter jejuni* and *Campylobacter* *coli* are the main causative agents of campylobacteriosis, posing a threat to public health worldwide (EFSA & ECDC, 2019). Fever, bloody diarrhea, headache and abdominal pain, nausea and vomiting are the main symptoms of campylobacteriosis in humans. Generally, the infection is self-limiting after 3-5 days, but in immunocompromised individuals it can spread into the bloodstream and become potentially lethal (Whitehouse et al., 2018). In severe cases, the antibiotic treatment is required, with macrolides and fluoroquinolones being the drugs of first choice (Mourkas et al., 2019). Campylobacteriosis is a mainly food-borne disease in which foods of animal origin, such as poultry meat, beef and pork, play a primary role (Sheppard et al., 2011).

Several studies showed the possibility of wildlife or environmental sources to act as reservoirs of *Campylobacter* infection (Sheppard et al., 2009a; Griekspoor et al., 2013; Cody et al., 2015; Atterby et al., 2018; Marotta et al., 2019; Marotta et al., 2020). In particular, these researchers focused on agricultural settings, especially on wild birds (Sheppard et al., 2009a; Griekspoor et al., 2013; Cody et al., 2015; Atterby et al., 2018; Marotta et al., 2019) small mammals (Sippy et al., 2012) and insects (Hald et al., 2004). However, there are little data on potential spill-over between livestock and wild ungulates (Navarro-Gonzalez et al., 2014). In pig farms, campylobacteriosis often leads to a significant decrease in animal productivity and consequent economic losses (Hansson et al., 2018).

Domestic pigs and wild boars belong to the same species (*Sus scrofa*) making them susceptible to the same pathogens (Ruiz-Fons et al., 2006; Ruiz-Fons et al., 2008). As a result, wild boar populations infected with *Campylobacter* could pose a threat to the pig industry. The Eurasian wild boar is widely distributed throughout most of Europe and in the past 50 years their numbers have increased to an estimatedpopulation of over 2.2 million wild boars (Massei et al., 2015; Meier and Ryser-Degiorgis, 2018). In Italy, it is the most widespread wild ungulate with a consistent presence along the country, due to its high prolificacy, favorable climatic conditions, and to the depopulation of Apennine and Alpine areas (Apollonio et al., 2010; Stella et al., 2018). Wild boars may contract *Campylobacter* from avian species, due to constant contact with soil contaminated with bird droppings (Waldenström et al., 2002; Humphrey et al., 2007; Epps et al., 2013). The increasing communities of wild boars in the anthropized areas as possible reservoirs of different *Campylobacter* species represent a growing challenge for public and veterinary health systems (Jones et al., 2013; Miller and Sweeney, 2013). Numerous studies showed that AMR is still very common in *Campylobacter* strains isolated from farmed animals in many European countries (EFSA & ECDC, 2019). In particular, high level of antibiotic resistance was shown to ciprofloxacin, nalidixic acid and tetracycline (EFSA & ECDC, 2019) followed, especially in *C. coli*, by resistance to macrolides and aminoglycoside antibiotic classes. Moreover, an alarming trend towards multidrug resistance (MDR), particularly among *C. coli*, was also detected (Luangtongkum et al., 2009; Pascoe et al., 2017; Mourkas et al., 2019). In this study, we aimed to evaluate the genotypic diversity of *Campylobacter* in wild boar and domesticated pig populations circulating in Italy and identify AMR genes in the two species investigated in order to understand the extent to which *Campylobacter* species are common, indicating a potential inter-species transmission.

## Material and Methods

### Bacterial Strains and Species Identification

A total of 238 *Campylobacter* strains isolated using the bacteriological ISO method 10272-1:2017 and stored at the microbial strain collection of the National Reference Laboratory for *Campylobacter* (NRL, http://www.izs.it/IZS/Eccellenza/Centri_nazionali/LNR_-_Campylobacter) were included in the study. The collection comprised 178 *Campylobacter* pig strains isolated from carcasses and from fecal content and 60 *Campylobacter* wild boar strains isolated from liver, muscle and faeces, in Italy between 2012 and 2019. The strains were cultured on Columbia blood agar plates in microaerobic atmosphere at 42°C for 48 h and DNA was extracted using Maxwell instrument (Promega Corporation, Madison, WI, USA) according to the manufacturer’s instructions and quantified using a Nanodrop Spectrophotometer (Nanodrop Technologies, Celbio Srl., Milan, Italy). After an initial phenotypic characterization, suspected colonies were confirmed as thermotolerant *Campylobacter* and identified to species level using a multiplex and a simplex PCR, as described previously (Wang et al., 2002; Marotta et al., 2019). Strains used as positive PCR controls were *C. coli* NCTC 11353, *C. fetus* ATCC 19438*, C. jejuni* ATCC 33291, *C. upsaliensis* NCTC 11541 and *C. lari* NCTC 11552.

### Sequence Analysis and Identification of Antibiotic Resistance Genes

Total genomic DNA was used to prepare sequencing libraries using Nextera XT Library Preparation Kit (Illumina, Inc., San Diego, CA, USA). The libraries were then sequenced using Illumina NextSeq 500 sequencer. Sequence reads (150-bp, pair-end) were demultiplexed and the adapters were removed. Subsequently the reads were trimmed with Trimmomatic tool (version 0.36) and *de novo* assembled using SPAdes version 3.11.1 with the “careful” option selected (Bankevich et al., 2012). The sequence reads generated in this study were deposited in NCBI Sequence Read Archive (SRA) in Bioprojects PRJNA638082 (https://www.ncbi.nlm.nih.gov/bioproject/PRJNA638082) and PRJNA638084 (https://www.ncbi.nlm.nih.gov/bioproject/PRJNA638084).

*C. jejuni* genome assemblies, were genotyped by MLST. The assemblies were also investigated for the genomic AMR traits.

The MLST profiles were assigned using a *C.jejuni/coli* task template MLST 7 loci, schema available at https://pubmlst.org/Campylobacter/accessible through in Ridom SeqSphere+ v. 6.0.2. Software (RidomGmbH, Münster, Germany). Italian MLST profiles were combined with MLST data of 1,121 pig isolates from Europe, downloaded from PubMLST (http://pubmlst.org/campylobacter/) and analyzed at the time of this analysis. MLST profiles were analyzed using the goeBURST algorithm implemented in PHYLOViZ, version 2.0 (Nascimento et al., 2017). Minimum spanning trees (MST) were created using default software settings.

The wgMLST analysis was performed in Ridom SeqSphere+ v. 6.0.2. The scaffolds were analyzed using two task templates: *C. jejuni/C. coli* cgMLST composed of 637 gene core gene targets and *C. jejuni/C. coli* accessory MLST composed of 958 accessory gene targets. Scaffolds that contained less than 90% good genome targets were excluded from the analysis. UPGMA tree was constructed by pairwise analysis of identified alleles, with missing targets ignored using default settings. The tree and associated metadata were visualized using iTol v5 (Letunic and Bork, 2006).

AMR genes were identified in silico using PointFinder v. 3.1.0 and ABRicate v. 0.8 (https://github.com/tseemann/abricate/) by querying the publicly available Comprehensive Antibiotic Resistance Database (CARD) (Jia et al., 2016; Zankari et al., 2017). Prokka v1.13 (Seemann, 2014) was used to annotate the assemblies and gyrA sequences were extracted applying the query_pan_genome function in Roary v3.12.0 (Page et al., 2015). gyrA genes were aligned using Uniprot UGENE v1.18.0 (Okonechnikov et al., 2012), from which the gene variants were identified. Only mutations in the quinolone resistance-determining region (QRDR) of gyrA were assessed to be the determinants of resistance, as only these loci have been linked with phenotypic resistance to quinolones. In addition, all the strains studied were deposited in PubMLST database (http://pubmlst.org/campylobacter) and the submissions ids are: BIGSdb_20200511094837_082196_21032, BIGSdb_20200511093337_081290_49754, BIGSdb_20200508081738_149794_16751 and BIGSdb_20200508080706_045922_07760.

### Antimicrobial Susceptibility

Antim icrobial susceptibility was tested by the broth microdilution method, using the Sensititre automated system (TREK Diagnostic Systems, Venice, Italy) following the manufacturer’s instructions. Briefly, colonies were subcultured on Columbia agar for 24 h and then seeded in Mueller Hinton Broth supplemented with blood (Oxoid, Basingstoke, UK). Then, they were dispensed into Eucamp2 microtiter plates (TREK Diagnostic Systems, Venice, Italy), with known scalar concentrations of the following antibiotics: ciprofloxacin (CIP) (0.12–16 μg/ml), erythromycin (ERY) (1–128 μg/ml), gentamicin (GEN) (0.12–16 μg/ml), nalidixic acid (NAL) (1–64 μg/ml), streptomycin (STR) (0.25–16 μg/ml), and tetracycline (TET) (0.5–64 μg/ml). The distribution % of MIC are reported in brackets. Following bacterial inoculation, the plates were incubated at 42°C in microaerobic atmosphere for 24 h, and then screened. The strains were classified as resistant (R), and susceptible (S) according to MIC breakpoints, by using Swin v3.3 Software (Thermo Fisher Scientific) in accordance with the epidemiological cutoff values (ECOFFs) as defined by EUCAST (European Committee on antimicrobial breakpoints) (www.eucast.org) to interpret their antimicrobial susceptibilities. *C. jejuni* strain NCTC 11351 was used as control. MIC breakpoints of resistance were > 0.5 μg/ml for CIP (*C.jejuni* and *C.coli)*, > 4 μg/ml for STR (*C.jejuni* and *C.coli)*, > 4 μg/ml for ERY (*C.jejuni)* and > 8 μg/ml (*C.coli)*, > 2 μg/ml for GEN (*C.jejuni* and *C.coli)*, > 16 μg/ml for NAL (*C.jejuni* and *C.coli)* and > 1 μg/ml for TET (*C.jejuni*) and > 2 μg/ml (*C.coli*). Details of the pig and wild boar isolates are summarized in **Supplementary Table 1**.

### Statistical Analysis

The antimicrobial resistance analysis was performed by means of a Chi-square statistic test. All values with P<0.05 were considered statistically significant (McHugh, 2013).

## Results

### Genus and Species Confirmation

We analyzed 178 *Campylobacter* strains isolated from carcasses (53.37%) and fecal content of pigs (46.62%), and 60 *Campylobacter* strains isolated from feces (83.33%), liver (10%), and muscle (6.67%) of wild boars (**Table 1**). *C. coli* was isolated in in 98.31% of pig and 91.66% wild boar strains, while *C. jejuni* was isolated in 1.68% and 8.33% of pig and wild boar strains, respectively (**Table 1**).

**Table 1:** Percentages of *Campylobacter* *coli* and *jejuni* isolated from pigs and wild boars.

	Carcass	Feces	Muscle	Liver
**Pigs (n=178)**	92 (51.68%) *C. coli* 3 (1.68%) *C. jejuni*	83 (46.62%) *C. coli*	–	–
**Wild boars (n=60)**	–	46 (76.66%) *C. coli* 4 (6.66%) *C. jejuni*	4 (6.7%) *C. coli*	5 (8.33%) *C. coli* 1 (1.66%) *C. jejuni*

### MLST Analysis of *C. coli* and *C. jejuni* Isolates

**Figure 1:**
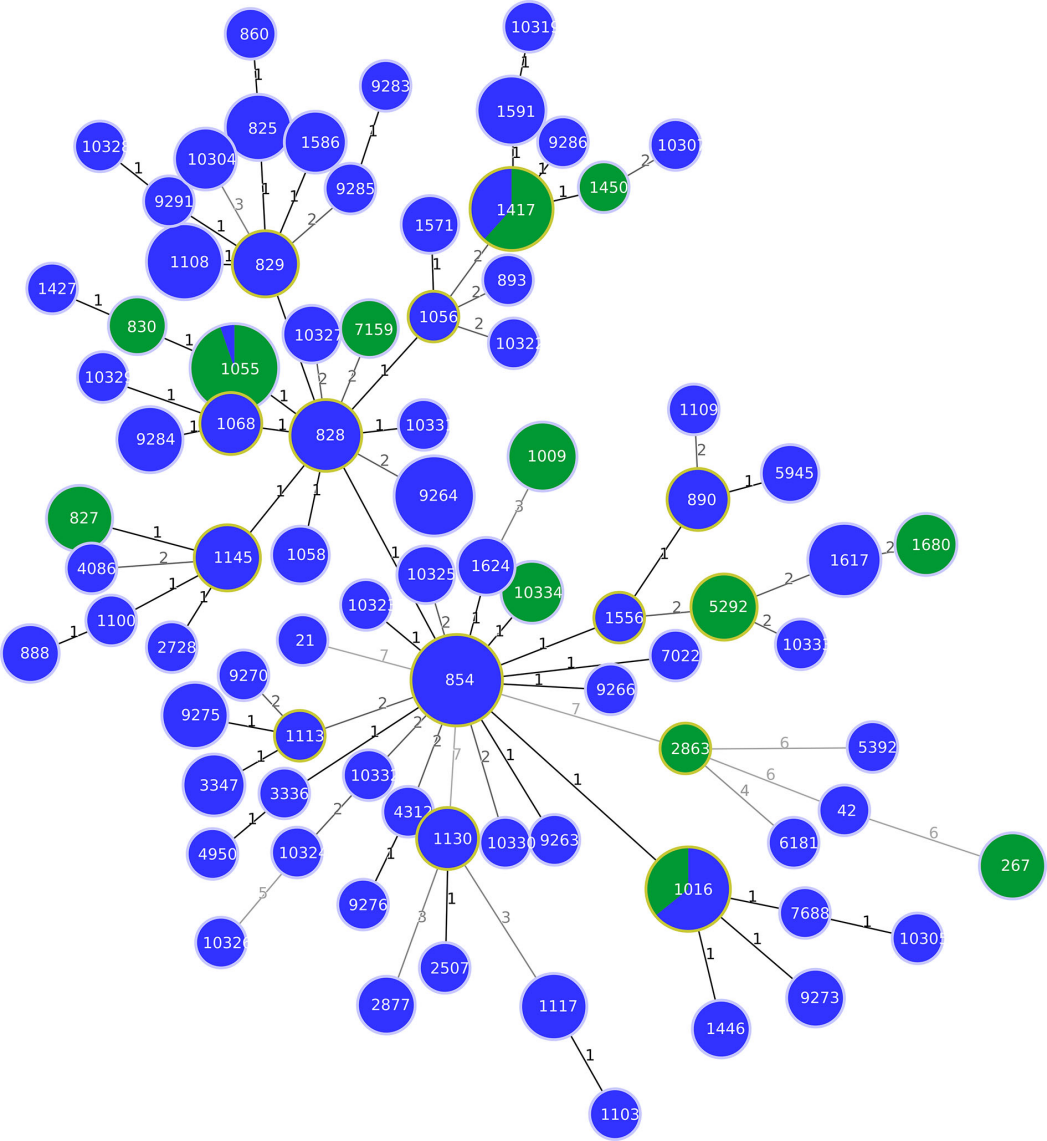
Minimum spanning tree (MST) generated for 238 Italian strains isolated from pigs and wild boars. The tree was generated using the goeBURST algorithm in PHYLOViZ software. The distance labels correspond to the number of discriminating alleles. The blue nodes correspond to pig isolates and the green nodes to wild boar isolates.

The MLST analysis showed 5 STs among the 8 C*. jejuni* strains studied (**Supplementary Table 1**). One ST (ST-10326) has not been described before in the PubMLST *Campylobacter* database (https://pubmlst.org/campylobacter/). The ST-10326, ST-42, ST-21 were assigned to *C. jejuni* strains isolated from 3 pigs, while ST-267 was assigned to 4 and ST-2863 to one wild boar *C. jejuni* strains (**Supplementary Table 1**). Regarding *C. coli*, 67 and 8 different STs were obtained from pigs and wild boars, respectively (**Supplementary Table 1**). Fifteen STs from pigs (ST- 10304, ST-10305, ST-10307, ST-10319, ST-10323, ST-10324, ST-10325, ST-10326, ST-10327, ST-10328, ST-10329, ST-10330, ST-10331, ST-10332, and ST-10333) and one ST from wild boars (ST-10334) were identified for the first time in this study (**Supplementary Table 1**). In particular, the novel STs contained one or more new allelic genes, and 12 novel alleles were found (aspA547, aspA548, aspA549, glnA754, gltA644, pgm1067, pgm1068, pgm1069, tkt824, tkt825, tkt826, uncA681). Fifty-five STs obtained from both hosts, belonged to the CC-828, only one ST isolated from one pig (ST-5392) belonged to CC-1150, and twenty-eight STs from pigs and wild boars did not belong to any known CC at the time of this analysis (**Supplementary Table 1**). The ST-1055 was the most prevalent ST that grouped 18 strains isolated from wild boars (30%). The second most prevalent ST was ST-1417 assigned to 8 strains isolated from wild boars (13.3%) (**Figure 1**). *C. coli* strains belonging to ST-854 were instead dominant in pigs (12.4%), followed by ST-9264 (6.18%). Out of 70 STs, 42 (60%) were obtained from pigs, and 2 STs out of the 11 STs (18.18%) isolated from wild boars, were represented by only one strain. Only three STs (ST-1016, ST-1055 and ST-1417) were shared between the two animal species (**Figure 1**). In detail, ST-1016 was represented by 14 C*. coli* strains (9 from pigs and 5 from wild boars); ST-1055 was represented by 19 C*. coli* strains (1 from pig and 18 from wild boars) and, finally, ST-1417 was represented by 13 C*. coli* strains (5 from pigs and 8 from wild boars). The MLST analysis with European pig isolates found a substantial number of STs (67) circulating only on Italian territory (**Supplementary Figure 1**). The STs most commonly shared with other European countries were: ST-854 and ST-828 shared with seven European countries (Scotland, Switzerland, Germany, UK, Portugal, Netherlands and Luxemburg), followed by ST-1016 shared with six European countries (Switzerland, Belgium, Scotland, UK, The Netherlands and Portugal). A total of 6 and 7 different STS were common with 3 and 2 other European countries, respectively, and 14 STs were shared with one other European country (**Supplementary Table 2** **).** The European countries with most STs shared with Italian isolates were Scotland (13 STs), Switzerland (10 STs) and Germany (9 STs).

### WgMLST Analysis of *C. coli*

**Figure 2:**
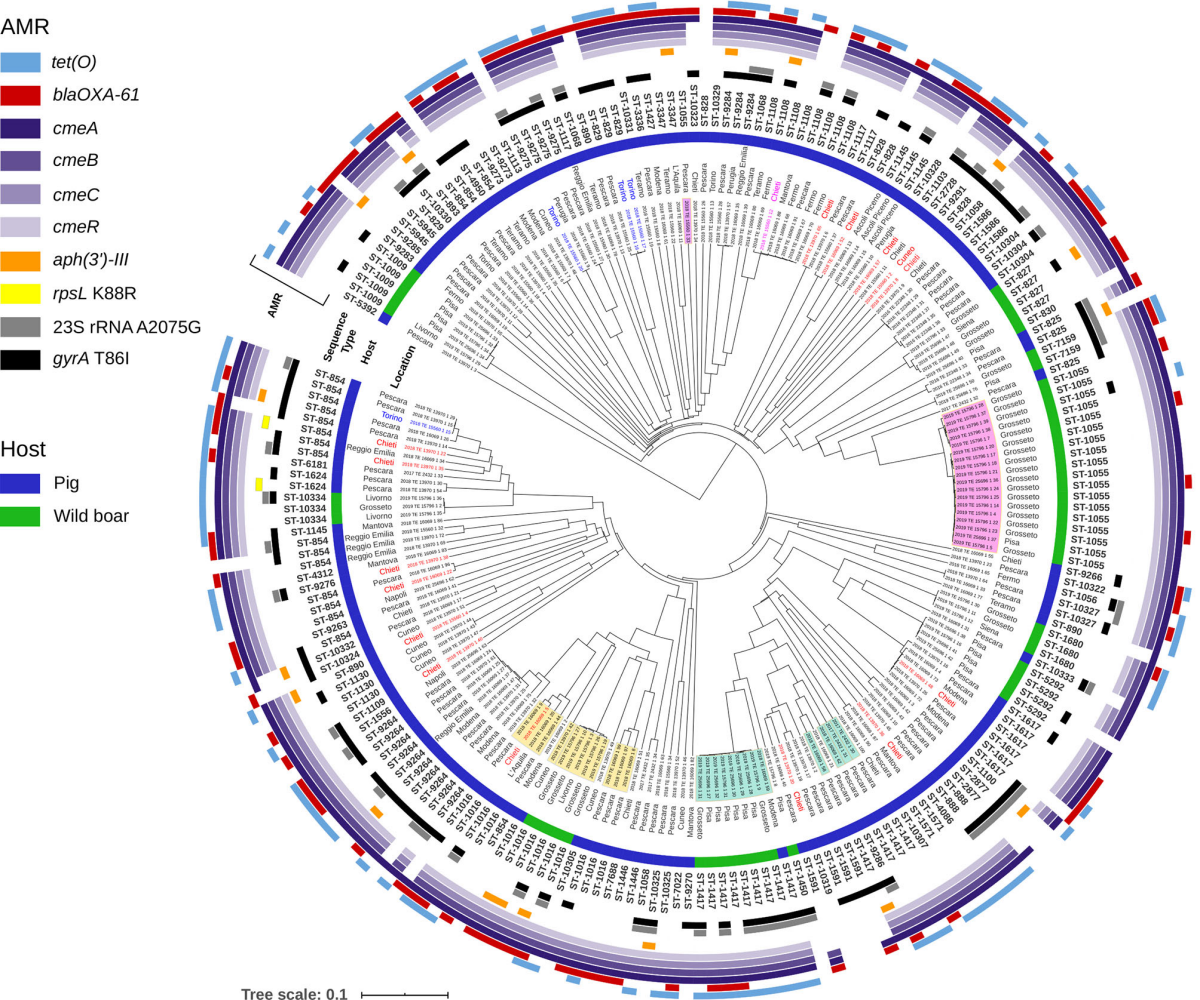
Phylogenetic tree generated for 213 strains of *C. coli* from Italy. The UPGMA tree was constructed based on wgMLST analysis results. The presence and allelic diversity of antimicrobial resistance genes substitutions in *C. coli* genomes are indicated. Strains isolated from domestic pigs are marked with blue color bar and from wild boar with green bar. The isolates highlighted in yellow, green and pink, strains obtained from the two different hosts and belonging to the same MLST sequence types. The isolates in red are of Hungarian origin, those in blue of Danish origin and the only one in fuchsia of French origin. The rest of isolates in black are of Italian origin.

The wgMLST analysis of 213 genomes of *C. coli* revealed wide diversity among the strains circulating in Italy (**Figure 2**). The maximum distance between the pair of wgMLST profiles was 583 genes. The strains isolated from domesticated pigs were scattered along most branches of the phylogenetic tree and few clusters of genetically closely related genotypes could be identified. Interestingly, even within these clusters, we did not observe clear geographic separation as they often contained strains isolated in two or more different locations. Similarly, *C. coli* isolates from wild boar, even though all collected in the Tuscany region, were divided into several separate lineages. The biggest cluster was found in Grosseto province and contained strains assigned to ST-1055. This sequence type was one of the three shared by both, *C. coli* strains from domesticated pigs and from the wild boarHowever, the isolate from the pig was distant from the wild boar ST-1055 complex by more than 400 genes demonstrating that ST determination was not sufficient to find real genetic connections between the strains. Moreover, we did not identify any clusters of closely related wgMLST profiles that contained strains from both the domesticated pig and the wild boar.

#### Antimicrobial Resistance Phenotypes

**Figure 3:**
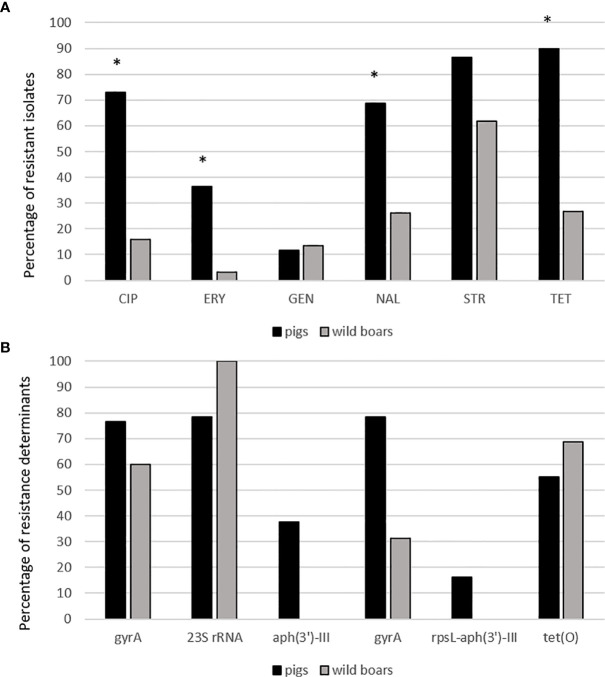
**(A)** Antibiotic resistance pattern between pig and wild boar isolates. CIP, ciprofloxacin, ERY, erythromycin, GEN, gentamicin, NAL, nalidixic acid, STR, streptomycin, TET, tetracycline. *statistically significant vs. wild boar isolates (*χ^2^*-test, p<0.01). **(B)** Percentages of resistance determinants between pig and wild boar antibiotic resistant isolates.

**Figure 4:**
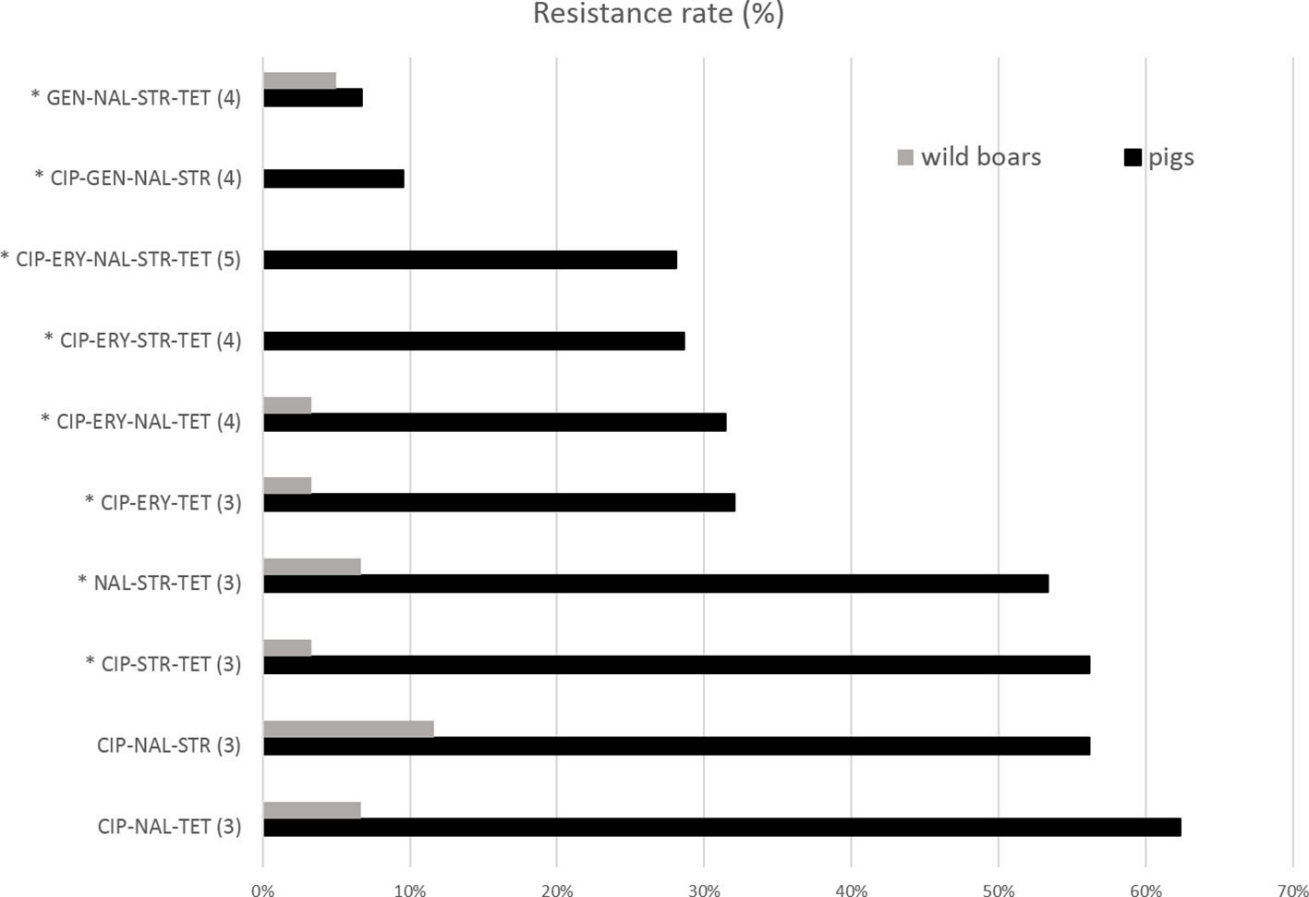
Percentages of resistant isolates to tested antibiotics among *Campylobacter* pig and wild boar isolates. CIP, ciprofloxacin, ERY, erythromycin, GEN, gentamicin, NAL, nalidixic acid, STR, streptomycin, TET, tetracycline. The antibiotic numbers are reported in parenthesis. *= MDR profiles.

The resistance levels of pig isolates to six antibiotics were compared to genomic resistance profiles of isolates of wild boar origin in **Table 2** and **Figures 3** and **4**. Statistically significantly higher levels of AMR in pig isolates in respect to wild boar isolates were observed for TET (89.9% vs 26.7%), CIP (73.1% vs 16%), NAL (68.9% vs 26%) and ERY (36.5% vs 3.3%) (Chi-square test; p<0.01). The MIC test revealed that 86.5% of pig and 61.6% of wild boar isolates were resistant to STR. Lower resistance levels were observed for GEN (11.6% for pig isolates; 13.5% for wild boar isolates) (**Figure 3**). MDR, considered as the resistance to at least three different classes of antibiotics (EFSA & ECDC, 2015), was very common (**Figure 4**). Strains isolated from pigs were more often found to display MDR than the strains from the wild boar. The most common MDR profiles were CIP-STR-TET (56% pig isolates; 3% wild boar isolates), followed by NAL-STR-TET (53% pig isolates; 7% wild boar isolates). CIP-ERY-TET was found in the 32% and 3% of pig and wild boar isolates, respectively, while CIP-ERY-STR-TET was present only in 29% of pig isolates (**Figure 4**).

**Table 2:** Comparison of genotypic and phenotypic resistance to antibiotics in *C. coli* isolated from Italian pigs and wild boars.

Antibiotic class	Antibiotics	Genes	Animals	No. of isolates with R phenotype^a^ (n=178)	No. of isolates with R genotype^b^	Concordance rate^d^
**Aminoglycosides**	Gentamicin (GEN)	*aph*(3’)-III	Pig	n=24	n= 9	37.5
			Wild boar	n=7	n=0	0
	Streptomycin (STR)	*rpsL-* *aph*(3’)-III	Pig	n=154	n=2; n=23	1.3–15
			Wild boar	n=37	n=0	0
**Beta-lactams** **^c^**	–	*OXA-61*	Pig	–	n=89	–
			Wild boar	–	n=27	–
**Fluoroquinolones/** **Quinolones**	Ciprofloxacin (CIP)/ Nalidixic acid (NAL)	*gyrA*	Pig	n=129; n=121	n=99; n=95	76.7–78.5
			Wild boar	n=10; n=16	n=6; n=5	60–31.2
**Macrolides**	Erythromycin (ERY)	*23S rRNA*	Pig	n=65	n=51	78.5
			Wild boar	n=2	n=2	100
**Tetracyclines**	Tetracycline (TET)	*Tet(O)*	Pig	n=160	n=88	55
			Wild boar	n=16	n=11	68.7
**Multidrug CmeABC efflux system and *cmeR***		*cmeA,cmeB,cmeC, cmeR*	Pig	–	n=153; n=132; n=130; n=129	–
			Wild boar	–	n=60; n=58; n=57; n=57	–

a Number of isolates expressing the resistance phenotype for the corresponding antibiotic;

b Number of isolates expressing the resistance phenotype for the corresponding antibiotic, that have the indicated gene;

c Antibiotic class does not tested for resistance phenotype;

d Concordance rate (%).

### Detection of Resistance Genes, Mutations, and Levels of Concordance

The genome assemblies of all *Campylobacter* were investigated for the genomic AMR genes, 23S rRNA and *gyrA*-associated point mutations and RpsL substitutions. The analysis revealed the presence of 7 AMR genes including: *tet(O)*, *cmeA*, *cmeB, cmeC, cmeR, OXA-61, aph(3’)-III*. The resistance genes for the corresponding antibiotic were observed in most but not in all resistant isolates. Regarding resistance to aminoglycosides, resistance traits associated with GEN and STR (*aph(3’)-III*) resistance were exclusively found in 9 and 23 pig *C. coli* resistant strains, respectively. RpsL substitution at amino acid 88, involved in STR resistance, was found in only two pig *C. coli* isolates. The concordance rate between the two types of resistances was of 37.5% and 16.3% (**Table 2**). Although we did not test resistance to beta-lactams antibiotics class phenotypically, we detected the *OXA-61* gene in the half of the pig and wild boar isolates. *Tet(O)* gene, conferring resistance to TET, was detected in 88 pig and 11 wild boar isolates resistant to TET. The concordance rate resulted, respectively, of 55% and 68.7%. The ERY resistant strains were screened for the presence of mutations in 23S rRNA gene. The A2075G mutation was identified in 51 and 2 isolates from pig and wild boar resistant isolates, showing a concordance rate of 78.5% and 100%, respectively. The *cmeA, cmeB, cmeC*, *and cmeR* genes, associated with efflux pump function, were present in almost all the strains. Finally, isolates resistant to fluoroquinolones and quinolones were screened for mutations in the *gyrA* gene. T86I mutation was detected in 99 and 6 pig and wild boar isolates with CIP resistance phenotype, showing a concordance rate of 76.7% and 60%, respectively, and in 95 and 5 pig and wild boar isolates with NAL resistance phenotype, showing a concordance rate of 78.5% and 31.2% (**Table 2**).

## Discussion

Here we presented a cross-sectional study on *Campylobacter* from Italian fattening pigs and wild boars using a multiplex approach that included antimicrobial susceptibility test, MLST, wgMLST, and genetic determination of AMR. The analyzed strains were representative of the Italian pigs and wild boars for the period 2012–2019. A high genomic diversity was observed among *C. coli* isolates in the Italian pig and wild boar populations, with 67 and 11 different STs within 175 and 55 analyzed isolates, respectively. These data are in line with other recent studies (Egger et al., 2012). In this study, MLST revealed the existence of the dominant *C. coli* CC-828 containing 76% of pig and wild boar isolates while the CC-1150 was detected only in one pig isolate. In addition, we observed that *C. coli* strains from pig and wild boar constituted two separate populations. Interestingly, only 3.7% (3/81) of STs were shared between pig and wild boar isolates. However, wgMLST analysis showed that pig isolates belonging to these three STs were genetically distant from the wild boar strains, demonstrating that ST determination was not sufficient to find real genetic connections between the strains of the two animals. In general, we did not identify any clusters of closely related wgMLST profiles that contained strains from both hosts suggesting that no exchange of *Campylobacter* spp. occurred between pigs and the wild boars, possibly due to the segregation of traditional pig farming and wild boar population. Interestingly, we noted that three pig strains (ST-829), isolated from pigs born in Denmark, had related wgMLST profiles, although were fattened in 2 different farms located in Pescara and Torino. Similarly, we showed several clusters in pigs with strictly related wgMLST profiles belonging to fattening farms located in different Italian regions. It is likely that fattening farms in Italy and in Europe may share the same feeder pig supplier, which would explainthe genomic relatedness observed in the distant farms. Comparison of our dataset with the strains obtained from *Campylobacter* MLST database revealed that *C. coli* population in Italian pigs and wild boars was different from other European countries. The *C. coli* strains featured with ST circulating only in Italy amounted for 82.7% (67/81) of the entire Italian collection, suggesting a geographical difference between the Italian and European populations. Furthermore, twenty STs were novel, likely representing geographically restricted clones, as reported also by other authors (Stone et al., 2013). Although the lack of WGS data hampered the verification of the genomic relatedness, it was surprising to observe a numerous STs shared between Italy and Scotland, indicating a possible internationally spread driven by the pig industry. However, a limitation of the study was the underrepresentation of *Campylobacter* isolates from wild boars in the PubMLST. As suggested in many studies we likely found several host-associated alleles that are present in *Campylobacter* (French et al., 2005; Miller et al., 2006; Litrup et al., 2007).

In this study, we revealed a clear separation between pig and wild boar *Campylobacter*, as shown by the presence of only three shared STs out of 83. It was also previously suggested that host preference or niche adaptation for certain STs play a role in acquisition and maintenance of specific clones in different host species (Schouls et al., 2003). Although our study did not allow us to draw conclusions on host association, it is likely that wild boars harbour *Campylobacter* STs that are rarely, if ever, transmitted to domestic pigs, possibly due to rare contact between the two hosts. Although wild boars are an environmentally destructive invasive species acting as a reservoir for zoonotic pathogens, our findings suggest that they might not be the primary source of infection of *Campylobacter* for traditional bio-secured domestic pig farms in Italy.

Despite the ban on the application of antibiotics as growth promoters in animal farms in the EU, *C. jejuni* and *coli* isolated from humans and animal sources show high levels of resistance to the most important antimicrobials used to treat campylobacteriosis (Castanon, 2007; EFSA & ECDC, 2019). As well as fluoroquinolones and tetracyclines, *C. coli* strains show a higher resistance to macrolide erythromycin and to aminoglycoside streptomycin, compared to *C. jejuni* (EFSA & ECDC, 2019). This is worrying because the use of fluoroquinolones, known to be the first-choice treatment for campylobacteriosis, has been recently shifted to erythromycin, against which *Campylobacter* resistance seemed to develop more slowly, in respect to fluoroquinolones-resistance (Lapierre et al., 2016). *Campylobacter* resistance mechanisms against the principal antibiotic classes are well known. Fluoroquinolone resistance is rapidly developed in *Campylobacter* strains because it requires only a single point mutation in *gyrA* gene (Luangtongkum et al., 2009). On the contrary, erythromycin resistance is due to specifics mutation in 23S rRNA and also depends on an rRNA methylation enzyme (*erm B*) (Wang et al., 2014). Tetracycline resistance is associated with the presence of *tet(O)* gene, encoding for a ribosomal protection protein (Sougakoff et al., 1987), while aminoglycosides resistance is due to several genes including *rpsL* and *aph(3’)-III* (Iovine, 2013; Zhao et al., 2016). *Campylobacter* is also known as a bacterium naturally resistant against Beta-lactams, (owning the ubiquitous gene *OXA-61*)used in combination with beta-lactamase inhibitors, when fluoroquinolones and macrolides are inefficacious (Griggs et al., 2009). Furthermore, among *C. coli*, which usually harbor AMR genes, a worrying trend towards MDR have been displayed. For all these reasons, *Campylobacter* has been categorized as a high priority pathogen on the list of bacteria for which new antimicrobials are urgently needed (WHO, 2017). In the present study, high levels of resistance to streptomycin, ciprofloxacin and tetracycline were detected in *C. coli* isolated from pigs, with resistance to streptomycin frequently found also among *C. coli* isolated from wild boars. Although the erythromycin resistance levels were lower, the existence of 36% of pig strains resistant to this antibiotic, which is the first-choice drug in the treatment of campylobacteriosis, is alarming. These resistance rates are in line with those reported by other European studies (García-Fernández et al., 2018; Di Donato et al., 2020).

In our study, we found a good correlation between phenotypic resistance to erythromycin, tetracycline, fluoroquinolones and quinolones and the presence of one or more resistance genes or nucleotide polymorphisms expected to confer resistance to the respective antimicrobials. For erythromycin, we found a correlation of 100% and 78.5% between the two types of resistances in pigs and wild boars, respectively. It is possible, that determinants of erythromycin resistance that were not analyzed in our study, such as mutations in L4 and L22 or in the regulatory region of CmeABC efflux pump, could be responsible for enhanced resistance in absence of mutations in 23S rRNA genes (Bolinger and Kathariou, 2017). For tetracyclin, the correlation varied between 68.7% and 55% of the presence of putative resistance genes and observed resistance phenotype respectively in pigs and wild boars. For fluoroquinolones and quinolones, the concordance rate varied between 77% and 45%, in pigs and wild boars. Discrepancies were found for rpsL mutation and the observed phenotype and for aminoglycosides, which could be explained with the existence of the efflux pump mechanisms or other unknown resistance mechanisms. These results suggest that, on one hand, the incidence of AMR in *C. coli* isolated from wild boars could be still considered low, showing that pigs, animals reared for food production, are much more exposed to antimicrobials. On the other hand, the results obtained show us the hazardous spread of AMR genes through the environment. A reassuring finding from our study was that *C. coli* isolated from wild boars have MDR profiles lower than 10%, in respect to MDR profiles of pigs, which were 5 times higher.

In conclusion, a rational and moderate use of antimicrobials, combined with a continuous monitoring of AMR bacteria spread in the environment, should be guaranteed to fight the increase in antibiotic resistance rates, extremely dangerous for human and animal health.

## Data Availability Statement

The raw data supporting the conclusions of this article will be made available by the authors, without undue reservation.

## Author Contributions

LDM and AA carried out the experiment. FM and AJ wrote the manuscript with support from GG. EDG, RN, and GG helped supervise the project. FM and EDG conceived the original idea. GG supervised the project. GDD, AJ, and FP analyzed the data and reviewed the manuscript. All authors contributed to the article and approved the submitted version.

## Funding

This work was supported by the Italian Ministry of Health, grant No IZS AM 07/17 RC and partially by grants of the University of Pisa (PRA_2018_56).

## Conflict of Interest

The authors declare that the research was conducted in the absence of any commercial or financial relationships that could be construed as a potential conflict of interest.

## Supplementary Material

Supplementary Figure 1 Minimum spanning tree (MST) generated for 1121 European and Italian strains isolated from pigs and wild boars. The tree was generated using the goeBURST algorithm in PHYLOViZ software. The distance labels correspond to the number of discriminating alleles. The blue nodes correspond to Italian isolates and the red nodes to European isolates.

Supplementary Table 1 List of pig and wild boar isolates with MLST and AMR genes profiles, phenotypic resistance and mutations.
