# Supplementary figures and images for: Genotyping and Antibiotic Resistance Traits in Campylobacter jejuni and coli From Pigs and Wild Boars in Italy
Source: Front Cell Infect Microbiol. 2020 Oct 15;10:592512. doi: 10.3389/fcimb.2020.592512 (PMC7593542; doi:10.3389/fcimb.2020.592512)

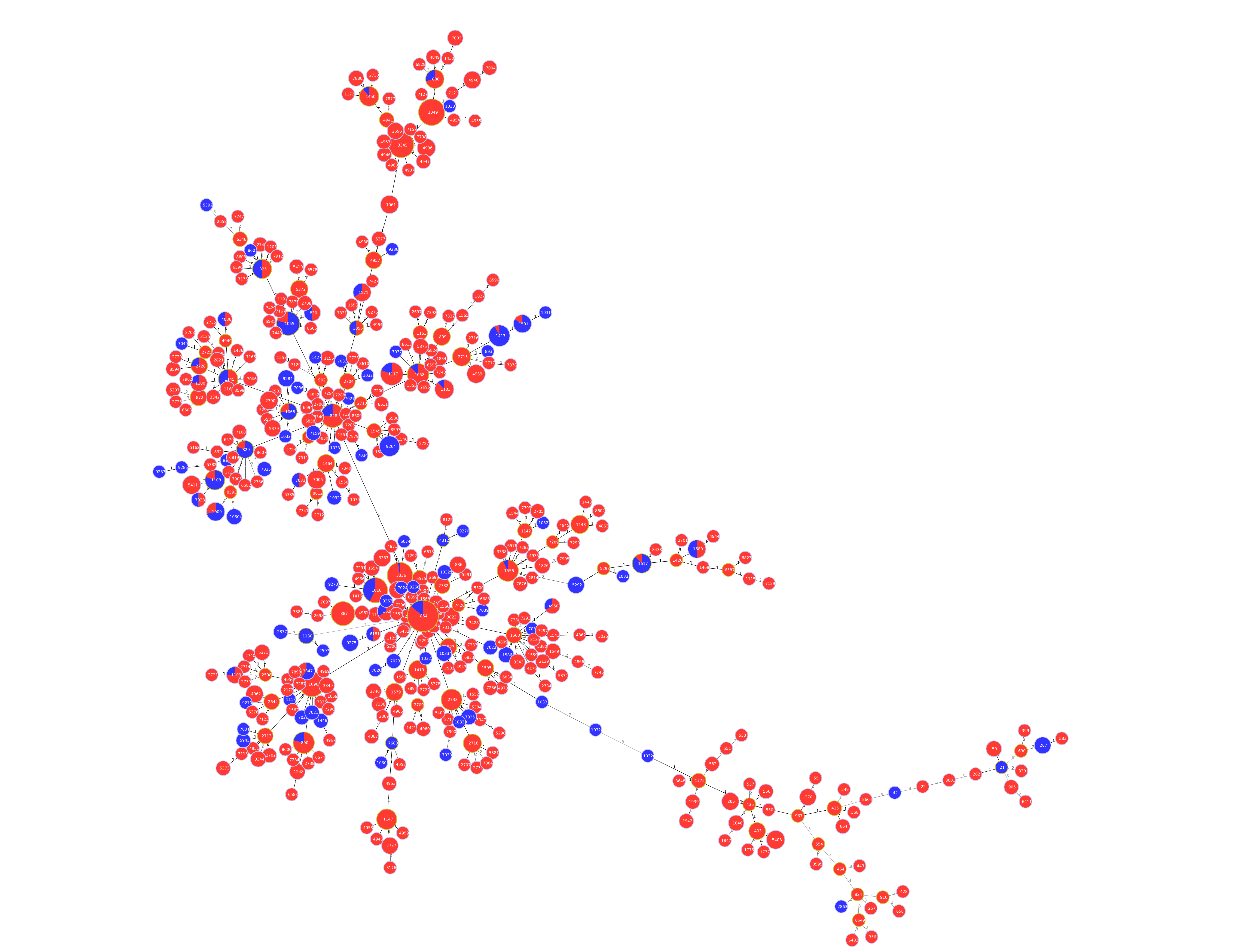

Supplement: Supplementary Figure 1 — Minimum spanning tree (MST) generated for 1121 European and Italian strains isolated from pigs and wild boars. The tree was generated using the goeBURST algorithm in PHYLOViZ software. The distance labels correspond to the number of discriminating alleles. The blue nodes correspond to Italian isolates and the red nodes to European isolates. [file Image_1.tif]
