# Supplementary material for: Genotyping and Antibiotic Resistance Traits in Campylobacter jejuni and coli From Pigs and Wild Boars in Italy
Source: Front Cell Infect Microbiol. 2020 Oct 15;10:592512. doi: 10.3389/fcimb.2020.592512 (PMC7593542; doi:10.3389/fcimb.2020.592512)
Supplement: Supplementary file 3 [file Table_2.docx]

**Supplementary Table 2.** List of STs isolated from pigs of European origin shared with Italian isolates.

| STs | Country | No. of countries |
| --- | --- | --- |
| 854-828 | Scotland, Switzerland, Germany, UK, Portugal, Netherlands and Luxemburg | 7 |
| 1016 | Switzerland, Belgium, Scotland, UK, The Netherlands and Portugal | 6 |
| 1055 | Scotland, The Netherlands and Denmark | 3 |
| 890 | Scotland, Portugal and Denmark | 3 |
| 1103 | Scotland, Germany and UK | 3 |
| 1058 | Scotland, Denmark and UK | 3 |
| 825 | Scotland, Switzerland and Spain | 3 |
| 1145 | Scotland, Spain and Finland | 3 |
| 888 | France and UK | 2 |
| 830-1680 | Germany and Switzerland | 2 |
| 1117 | Germany and Denmark | 2 |
| 1109 | UK and Scotland | 2 |
| 1624 | Switzerland and Scotland | 2 |
| 1108 | Germany and Poland | 2 |
| 1617-1591-1450 | Scotland | 1 |
| 3347-1009-1100 | Switzerland | 1 |
| 6181-1571-1068 | Germany | 1 |
| 829-7033 | UK | 1 |
| 1417 | Spain | 1 |
| 1056 | Luxemburg | 1 |
| 4086 | France | 1 |
